# Comparative Analysis of Fracture Resistance of Endodontic Sealer Types and Filling Methods

**DOI:** 10.3390/ma18010040

**Published:** 2024-12-26

**Authors:** Yun Song, Kee-Deog Kim, Bock-Young Jung, Wonse Park, Nan-Sim Pang

**Affiliations:** Department of Advanced General Dentistry, College of Dentistry, Yonsei University, Seoul 03722, Republic of Korea

**Keywords:** bioceramic sealer, fracture resistance, mineral trioxide aggregate, resin-based sealer, root canal obturation, root fracture

## Abstract

With the advent of bioceramic sealers, sealers have become a more important filling material for endodontic treatment. When a solid sealer, rather than an elastic gutta-percha, occupies a significant portion of the root canal, it is unclear whether the tooth structure will be strengthened to withstand stress or whether the increased stiffness will transmit the load directly to the entire root, potentially causing root fracture. This study compared the fracture resistance and fracture patterns of roots filled with various root canal sealers, including bioceramic sealers, and each corresponding filling technique. Five groups (*n* = 10) were tested, including no endodontic treatment, no canal obturation, resin-based sealers with continuous-wave compaction, bioceramic sealers with single-cone technique, and mineral trioxide aggregate (MTA) orthograde obturation. The specimens were radiographed to assess the filling quality. After embedding the specimens in acrylic resin, fracture resistance was tested by a universal testing machine, and fracture features were examined microscopically. The results showed that the bioceramic sealer group using the single-cone technique had the highest fracture strength. Radiographic analysis revealed that achieving void-free filling was more difficult with MTA orthograde obturation compared to resin-based and bioceramic sealer groups. No significant variation in fracture features was observed across the groups.

## 1. Introduction

The long-term success of endodontic treatment is affected by microleakage from obturation materials for various reasons [1,2,3,4]. Gutta-percha has a lower modulus of elasticity than dentin, making the use of additional sealers necessary. However, these sealers can exhibit volume shrinkage due to dissolution over time. Conventional root canal-filling techniques attempt to minimize these volume changes by compacting the soft gutta-percha and applying a thin layer of root canal sealers [4].

Removing intracanal hard tissue during root canal treatment may weaken the tooth structure, increasing the risk of root fracture [5]. Vertical root fractures occur more frequently in previously treated teeth than in vital teeth [6,7,8]. The quality and quantity of the remaining tooth structures after root canal treatment affect the survival and fracture resistance of the teeth [9]. Previous studies have suggested that root canal preparation can increase the incidence of dentin defects such as microcracks [10,11]. Moreover, excessive force applied during root canal obturation can increase the risk of vertical root fracture [7]. The application of a spreader in the lateral condensation filling technique produces a wedging effect on the tooth. The vertical-condensation filling technique applies a considerable vertical load on the teeth. Subsequent stress loads, such as occlusal pressure, may further damage the weakened tooth structure. Therefore, various sealers and root canal obturation methods have been developed to reinforce lost tooth structures and ensure a complete seal [12].

Epoxy resin-based sealers have a higher bond strength than zinc oxide eugenol, glass ionomer, and calcium hydroxide-based sealers [13]. They are favored for their easy handling, good sealing properties, low solubility, and compatibility with gutta-percha and dentin surfaces [14]. Additionally, resin-based sealers do not show relevant chemical or physical changes after thermal treatment, making them ideal for warm vertical filling techniques or continuous-wave compaction techniques [15].

Mineral trioxide aggregate (MTA) is a biocompatible material that can be used in hydrophilic settings and induces hard tissue formation. MTA was originally used for root perforation repair and retrograde root filling [16]. In vitro studies have shown that MTA has lower cytotoxicity and better sealing ability than IRM, super-EBA, and amalgam [17,18,19]. Because of the excellent physical properties of MTA, some dentists have recently attempted to use it for orthograde canal filling to create a monoblock integrated with the root canal. However, the difficulty in manipulating MTA can cause voids during canal obturation and may not ensure complete root canal sealing [20]. Although MTA was developed as an injectable type sealer to penetrate into the root canal, it is difficult to expect the same physical properties as MTA unless it is pure MTA [21].

The drawbacks of using MTA, such as difficult handling and long setting time, have led to the development of calcium-silicate cement [22,23,24]. Calcium silicate-based sealers are a type of bioceramic sealers that are gaining popularity due to the convenience of the single-cone filling technique using pre-mixed sealers. They are known to have properties such as chemical stability, biocompatibility, radiopacity, hydrophilicity, flowability, and slight volume expansion [25]. In addition, this single cone filling technique does not require the force to compress gutta-percha within the root canal, which can reduce root damage caused by excessive force. However, compared with other canal obturation methods, the single cone filling technique using bioceramic sealer has a higher sealer-to-gutta-percha ratio, and the physical property of this sealer may directly affect the fracture resistance of the obturated root. When a solid sealer, rather than an elastic gutta-percha, occupies a significant portion of the root canal, it is unclear whether this strengthens the tooth structure against stress or whether the increased stiffness directly transfers the load to the entire root, potentially causing root fracture. In addition, the hygroscopic expansion of bioceramic sealer may cause microleakage and cracks in the root [26].

Previous studies have examined the effects of different sealing materials on fracture resistance using the same obturation techniques [27,28,29] or different obturation techniques with the same sealing materials [7,30]. However, few studies have examined how different combinations of sealing materials and obturation techniques actually used in dental clinics affect tooth fracture resistance [31]. Some dental practitioners have suggested monoblock formation by orthograde canal filling with MTA [12], but few studies have examined whether MTA or bioceramic sealers actually reinforce the remaining tooth structures [5].

In this study, we aimed to comparatively evaluate the fracture resistance and patterns of roots obturated with various root canal sealers, including bioceramic sealers, and their corresponding canal filling techniques using in vitro methods.

## 2. Materials and Methods

### 2.1. Teeth Selection and Disinfection

Fifty non-carious mandibular premolars with similar buccal-lingual and mesial-distal dimensions were selected for this study. All teeth were immersed in 5% sodium hypochlorite (NaOCl) solution for 30 min after extraction and then stored at 4 °C in 0.1% thymol solution (Sigma-Aldrich, St. Louis, MO, USA). Preoperative radiography and an optical microscope (OPMI Pico; Carl Zeiss AG, Jena, Germany) were performed to confirm that none of the teeth had root caries, crack lines, calcified canals, open apices, or fractures.

### 2.2. Specimen Preparation

**Figure 1:**
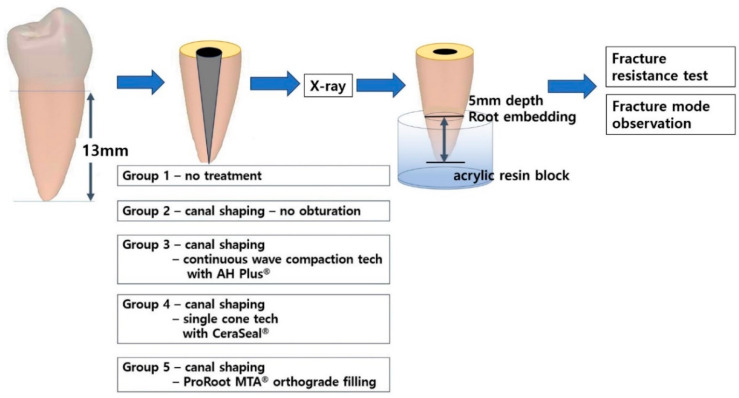
A scheme of the entire experimental procedure.

Fifty tooth samples were randomly assigned to five experimental groups (*n* = 10 each). A schematic representation of the grouping and the entire procedure is shown in Figure 1. The chemical composition of the sealers and filling material used in each group is summarized in Table 1.

All teeth were decorated to a standard length of 13 mm from the apex using a diamond bur. Group 1 did not undergo any instrumentation or obturation. Coronal access was performed using a #245 bur (excluding group 1, negative control). The working length was standardized by subtracting 1 mm from the root canal length. ProTaper Gold rotary Ni-Ti files (Dentsply Maillefer, Ballaigues, Switzerland) were used to shape the root canals. The files were used sequentially up to F3 at 250 rpm. The canals were irrigated with 1 mL of 5% NaOCl after each shaping step. Final canal irrigation was performed using 17% ethylene diamine tetra acetic acid (EDTA, DIRECTA AB, Stockholm, Sweden), 5% NaOCl, and normal saline.

Roots in Group 2 were dried with paper points but were not obturated (positive control). Roots in Group 3 were obturated with F3 ProTaper gutta-percha points (Dentsply Maillefer, Ballaigues, Switzerland) and AH Plus^®^ sealers (Dentsply, Charlotte, NC, USA) using a continuous-wave compaction technique. The gutta-percha was cut at a working length of 5 mm and back filled. Roots in Group 4 were obturated with F3 ProTaper gutta-percha points and CeraSeal^®^ (MetaBioMed, Chungju, South Korea) using a single-cone technique. Roots in Group 5 were obturated with ProRoot MTA^®^ (Dentsply Maillefer, Ballaigues, Switzerland) using an MTA messing gun and hand plugger. One dentist performed all root canal treatments.

### 2.3. Radiographic Evaluation for Canal Obturation

After canal obturation of groups 3, 4, and 5, post-obturation radiographs were obtained to ensure that the canal was adequately filled without voids. All specimens were radiographed buccolingually and mesiodistally to assess the quality of root canal obturation, particularly to confirm seal in the apex (5 mm).

### 2.4. Fracture Resistance Test

All specimens were stored at 37 °C and 100% humidity for 2 weeks to allow the materials to set completely. Subsequently, 5 mm of each root was embedded vertically into an acrylic block (autopolymerizable acrylic resin, Lang Dental, Wheeling, IL, USA) with a diameter of 15 mm and height of 13 mm to simulate the alveolar bone and provide a stable base for the fracture resistance test. All samples were mounted on a universal testing machine (Instron, Norwood, MA, USA) for fracture resistance testing. A ball-tip indenter (5 mm diameter) was used to apply a vertical load at a speed of 1 mm/min until the roots fractured. A fracture was determined when the load exhibited an instantaneous drop.

The load of the fracture resistance in Newtons was converted to mega-Pascals using the following formula:

MPA (N/mm^2^) = Maximum load in Newtons (N)/(π/4) × (Ø 5 mm)^2^

### 2.5. Evaluation of Fracture Modes of the Experimental Groups

**Figure 2:**
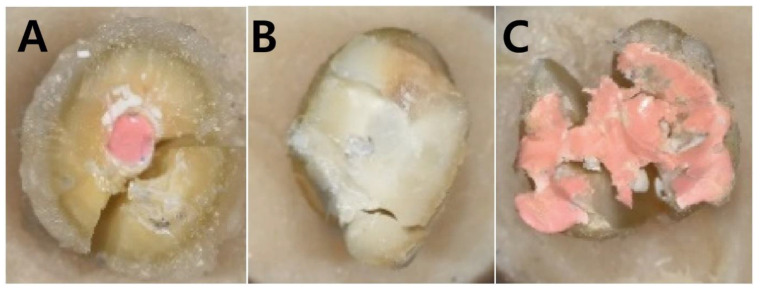
Evaluation of fracture modes: representative images were obtained from each group at 12.5× magnification. (**A**) adhesive failure: fracture of the interface between root canal sealer and dentin; (**B**) cohesive failure: fracture within canal sealer or dentin excluding the dentin-sealer border; (**C**) mixed-type fracture: destruction of canal filling material and dentin, including the dentin-sealer border.

All experimental groups were examined under an optical microscope (OPMI Pico; Carl Zeiss AG, Jena, Germany) with 12.5× and 60× wide-field eyepieces after the fracture resistance test. The failure type (adhesive or cohesive) of the sealing material to the canal wall was also evaluated. The fracture line passing through the border between the dentin and the root canal filling material was considered an adhesive failure. The fracture line that did not pass through the border between the dentin and the filling material was considered a cohesive failure. Multiple fracture lines, including both failure types, were considered mixed-type fractures (Figure 2). A complete fracture of the coronal tooth structure was also considered a mixed-type fracture. Groups 1 and 2 were excluded from the failure mode analysis because they did not undergo canal obturation.

### 2.6. Statistical Analysis

The normality of data distribution was evaluated using the Shapiro–Wilk test, followed by the homogeneity of variances using Levene’s test. Statistical analyses were performed using analysis of variance (ANOVA), least significant difference test, post hoc multiple comparisons, and Duncan’s test. Statistical significance was set at *p* value *<* 0.05. Fisher’s exact test was used to analyze the failure modes (*p* < 0.05). All statistical analyses were conducted using software SAS v.9.2 (SAS Inc., Chicago, IL, USA).

## 3. Results

**Figure 3:**
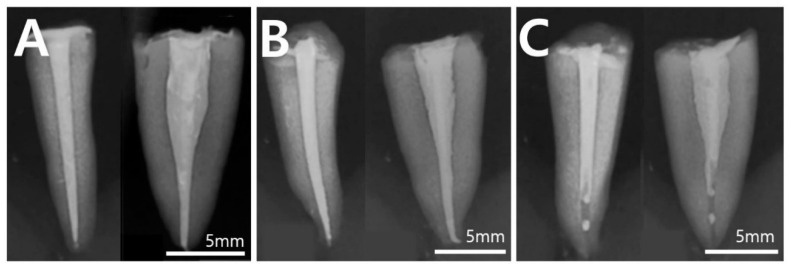
Representative post-obturation radiographs of groups 3, 4, and 5 (buccolingual view, mesiodistal view): (**A**) AH Plus^®^ with continuous wave compaction technique group 3; (**B**) CeraSeal^®^ with single-cone technique group 4; (**C**) ProRoot MTA^®^ with monoblock obturation technique group 5.

Examination of post-obturation radiographs showed that adequate filling without voids was more challenging when using MTA and the monoblock obturation technique (Figure 3). The MTA-filled group showed a dense filling quality in the coronal and middle parts, but apical underfilling and cavities were observed in all samples within 5 mm of the apical part. Roots filled with resin-based and bioceramic sealants showed more uniform root filling density than roots filled with MTA, without voids extending to the apical portion in most cases.

**Figure 4:**
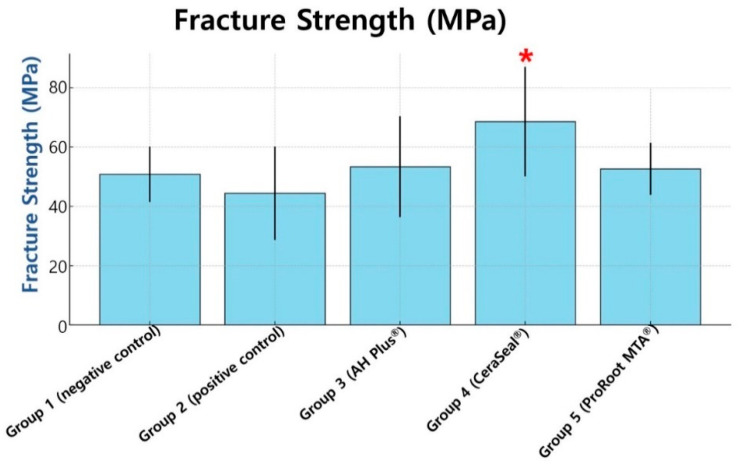
Mean of the fracture strength (MPa) of each group. Error bars present the standard deviations. A red asterisk (*) indicates a statistically significant difference compared to other groups (*p* < 0.05).

Table 2 and Figure 4 present the means and standard deviations of the fracture resistance strength in each group. Comparison of fracture resistance between the five groups using a one-way ANOVA test at the 5% level of significance showed that significant differences were observed between the five groups (*p* < 0.05, Table 3).

The bioceramic sealer prepared using the single-cone technique exhibited the highest fracture strength (68.6 MPa), with a significant difference (*p* < 0.05). The resin-based sealer (AH Plus^®^, 53.4 MPa) and ProRoot MTA^®^ (52.7 MPa) showed a higher mean force than the negative control group (group 1, 50.4 MPa), with no significant difference between them. The negative control group showed a higher fracture strength (50.8 MPa) than the positive control group (44.4 MPa), but the difference was insignificant.

A close examination of these samples under an optical microscope revealed that the resin-based sealer (AH Plus^®^) had more adhesive failures and mixed failures involving the dentin-filling material interface than cohesive failures not involving the interface (adhesive failure 50%, mixed failure 30%, cohesive failure 20%). In contrast, bioceramic sealers showed more cohesive failures (50%) than mixed failures (30%) and adhesive failures (20%). ProRoot MTA^®^ exhibited similar frequencies of adhesive, cohesive, and mixed failure modes (30%, 40%, and 30%, respectively; Table 4). However, none of these showed significant differences.

## 4. Discussion

Mandibular premolars were selected for this study because of their similar morphology. Cleghorn et al. showed that most mandibular premolars were single-rooted (99.6%), and anatomical variations were rare [29]. This method allowed for uniform load distribution and easier control of other variables between the experimental groups. However, in this study, we used only 50 mandibular premolars from various age groups because of the limited number of teeth available. The potential increase in fatigue and dentin microstructure changes in the premolars of older teeth may have affected the fracture resistance of teeth regardless of the filling materials or filling techniques [31]. In addition to age-related dentin changes, some limitations, such as clinical variability in the pretreatment status of the teeth, may affect the generalizability of the results. Compared with the infected dentin that dental practitioners typically encounter when providing root canal treatment to patients, all samples in this study were non-carious with sound dentin, which may produce different fracture resistance values.

In the present study, the positive control group (canal shaping, no obturation) showed a lower fracture resistance strength than the negative control group (no shaping, no obturation). This result indicated that canal shaping weakened the tooth structure, aligning with the findings of the previous in vitro studies [32,33,34]. In addition, chemical irrigation induces dentin dehydration, which reduces the elastic modulus, flexural strength, and microhardness of the root dentin [35,36]. Thus, it is important to select root canal filling materials and methods that can strengthen the remaining tooth structure. In this study, the MTA monoblock, resin-based sealer, and bioceramic sealer groups showed higher fracture resistance than the negative and positive control groups.

Prado et al. showed that the group using the continuous wave compression technique strengthened teeth and significantly increased fracture resistance compared to the unfilled positive group, but showed less resistance than the negative control group [37]. In contrast, Telli et al. suggested that the warm vertical compaction procedure does not cause premature root fractures when skillfully performed [38]. Although the difference was insignificant, this study showed that the fracture resistance of the AH plus^®^ sealer-continuous wave compaction group was higher than that of the negative and positive control groups. AH plus^®^ sealer, due to its creeping capacity and long setting time, flows into the canal and penetrates deeply into micro-irregularities, thereby increasing mechanical interlocking with the dentin in the root canal [13]. This leads to high bonding with the dentin on the root canal wall, and this bonding strength is further increased when the sealer penetrates deeper into the dentin wall by the pressure of the continuous-wave compaction method. Therefore, it is presumed that the use of AH plus^®^ sealer, a highly adhesive sealer in continuous-wave compaction technique, can offset the risk of root fracture, ultimately resulting in higher fracture resistance than the positive and negative control groups in this study. However, when a strong external force was applied, resin-based sealers and continuous wave compaction groups finally showed more adhesive failure than cohesive failure. The obturation material and the root canal wall did not become a single unit as a monoblock, and adhesive failure was observed at the sealer-dentin interface.

The bioceramic sealer group using the single-cone technique showed the highest fracture resistance strength among the experimental groups, with a significant difference (*p* < 0.05). Bioceramic sealers form hydroxyapatites and chemically bond with root dentin, which improves the fracture resistance of the root [39]. Calcium silicate of CeraSeal^®^ creates a tag-like structure at the calcium silicate-dentin interface. This hydroxyapatite recrystallization can reduce marginal gaps and increase cement retention. Calcium silicate-based sealants are micromechanically bonded to dentin, reducing interfacial gaps [40]. In addition, unlike resin-based sealers, bioceramic sealers, which are hydrophilic and have a low surface contact angle, easily spread on the root canal wall without external pressure and form a good seal through micro-mechanical interlocking [41]. Bioceramic sealer has a higher dentinal tubule penetration rate with an average particle size of 0.2 µm than the AH plus sealer, which contains 8 µm calcium tungstate particles and 1.5 µm zirconium oxide particles [42]. These result in the root-reinforcing effect of the bioceramic sealer and more cohesive failure than adhesive failure patterns. In other studies, the bioceramic-coated cones may further enhance the fracture resistance [43]. When GP cones coated with mini particles of calcium silicate as a sealer were used, a stronger bond occurred when they came into contact with the bioceramic sealer, and the GP cone, sealer, and root canal wall formed a monoblock as one unit, strengthening the tooth root. Although the bioceramic-coated cone was not used in this study, the expansion characteristic of bioceramic sealer also had an effect on the highest fracture strength. Water sorption of bioceramic sealers promotes slight volume expansion and thus sealing ability [44]. Concerns that hygroscopic expansion of the bioceramic sealers would increase the risk of root fracture have been disproved by their high fracture resistance. Cimpean et al. showed similar results, where cohesive failure modes were predominant in root canals filled with bioceramic sealers. In contrast, adhesive failure modes were predominant in those filled with resin-based sealers [45].

Previous studies have already revealed that the single-cone technique is a factor that increases fracture resistance [27,34]. In experiments that applied various root canal filling methods using the same sealer, the single-cone technique showed higher fracture resistance than warm vertical condensation and lateral compaction in most cases, and it was mentioned that the wedge effect of the spreader was a major cause of the increased risk of fracture. When using single-cone technique with various sealers, bioceramic sealer achieved the highest fracture resistance; thus, bioceramic sealer has been found to be the most suitable sealer for single-cone technique to date [34].

In contrast, the ProRoot MTA^®^ monoblock obturation technique showed the lowest fracture resistance among the experimental groups. Pure MTA orthograde filling has been used for apical plug formation of immature permanent teeth or pre-operative orthograde filling followed by root-end resection [46,47]. Recently, MTA sealer, one of the bioceramic sealers, has been used for orthograde filling using GP cone [21]. However, various products have been released that seal the root ends by injecting MTA cement into the root canal without GP cones [47,48]. Through various experiments, they have shown sufficient sealing ability similar to conventional root canal treatment [21,48,49]. In addition, it was expected that the monoblock, in which MTA cement and root dentin form a single unit without separation of the interface, would strengthen the root and increase resistance to root fracture [49]. However, research on root reinforcement of MTA monoblock was insufficient. The purpose of this study was to investigate the root-strengthening effect rather than the root-sealing effect. Therefore, pure MTA, which has better properties than MTA sealer for orthograde filling, was used, but the expected root-strengthening effect was not observed. The low fracture resistance of the MTA monoblock obstruction group in this study may have been due to poor filling quality caused by difficulties in MTA manipulation. This difficulty in the clinical manipulation of MTA may cause voids when root canals are obturated [21]. This makes it difficult to obtain a perfect seal within the root canal and expect a root-reinforcing effect. ProRoot MTA^®^ showed similar adhesive and cohesive failure modes, though it was insignificant. As a result of not being able to create an ideal monoblock with MTA, it was found that 60% of MTA cases showed fracture at the boundary between the root canal dentin wall and the MTA filling material (adhesive failure, mixed type fracture). This is higher than that of bioceramic sealer (50%), although it is not significant.

Compared with studies using fractures under a continuous vertical load, further studies using the thermocycling process and chewing simulation of roots may provide different results. Volume shrinkage and dissolution of sealers over time, dentin aging, and other obturation materials require further examination. Larger sample sizes and long-term clinical studies are required to evaluate the fracture resistance of various root canal sealers and determine the corresponding root canal filling techniques.

## 5. Conclusions

Based on the findings of this in vitro study, the following were concluded:

1. Root canal obturation increases the fracture resistance of canal-shaped roots.
2. The combination of the bioceramic sealer and the single-cone filling technique resulted in the highest fracture resistance, compared with the resin-based sealer with the continuous-wave compaction and MTA monoblock obturation technique.
3. MTA using the monoblock obturation group revealed root filling with voids on post-obturation radiography. Its fracture resistance was lowest among the filled groups, although the difference was insignificant.
4. Cohesive failure pattern was most prevalent in the bioceramic sealer-single cone technique group. However, there was no significant difference in fracture pattern frequency across all groups.

## Figures and Tables

**Table 1:** Endodontic sealers used in the study.

Material (Lot Number)	Classification	Composition	Manipulation	Manufacturer
AH Plus^®^ (2204000392)	Epoxy resin-based sealer	Epoxide paste: diepoxide, calcium tungstate, zirconium oxide, aerosol, pigment Amine paste: 1-adamantane amine, N,N′-dibenzyl-5-oxa-nonandiamine-1,9, TCD-Diamine, calcium tungstate, zirconium oxide, aerosol, silicone oil	2 paste type	Dentsply
CeraSeal^®^ (CSL2112281)	Bioceramic sealer (calcium silicate-based sealer)	Calcium silicate, zirconium oxide, thickening agents	Pre-mixed syringe	MetaBioMed
ProRoot MTA^®^ (9001766)	MTA filling material	bismuth oxide (19.8%), tricalcium silicate (51.9%), dicalcium silicate (23.2%), calcium dialuminate (3.8%), and calcium sulfate dehydrated (1.3%)	Powder (+sterile water)	Dentsply

**Table 2:** Mean value and standard deviation (SD) of fracture strength.

Groups	Fracture Strength (N)		Fracture Strength (MPa)		Significance *
	Mean	SD	Mean	SD	
Group 1 (Negative control)	997.6	±184.4	50.8	±9.4	A
Group 2 (Positive control)	872.1	±310.7	44.4	±15.8	A
Group 3 (AH Plus^®^)	1048.6	±333.8	53.4	±17.0	A
Group 4 (CeraSeal^®^)	1345.6	±362.5	68.6	±18.5	B
Group 5 (ProRoot MTA^®^)	1034.7	±171.8	52.7	±8.8	A

* The same letters in superscript show no significant differences determined by Duncan test.

**Table 3:** Comparison of fracture resistances between five groups using one-way ANOVA.

Scheme	Sum of Squares	Df	Mean Square	F	*p* Value
Between groups	2523.815	4	630.954	3.015	0.0031
Within groups	9415.710	45	209.238		
Total	11,939.525	49			

**Table 4:** Distribution of failure modes of each obturated root canal after the fracture resistance test (%).

Groups	Adhesive Failure	Cohesive Failure	Mixed Type Fracture	Total
Group 3 (AH Plus^®^)	5 (50)	2 (20)	3 (30)	10 (100)
Group 4 (CeraSeal^®^)	2 (20)	5 (50)	3 (30)	10 (100)
Group 5 (ProRoot MTA^®^)	3 (30)	4 (40)	3 (30)	10 (100)

## Data Availability

The data presented in this study are available upon reasonable request from the corresponding author due to privacy.

## Institutional Reviews Board Statement

This article does not contain any studies with human participants or animals performed by any of the authors. This study was approved by the Institutional Review Board of Yonsei University Dental Hospital, Seoul, Korea (No. 2-2021-0007).

## References

[1] Zhou H.-m., Shen Y., Zheng W., Li L., Zheng Y.-F., Haapasalo M. (2013). Physical properties of 5 root canal sealers. J. Endod..

[2] Jafari F., Jafari S. (2017). Importance and methodologies of endodontic microleakage studies: A systematic review. J. Clin. Exp. Dent..

[3] Muliyar S., Shameem K.A., Thankachan R.P., Francis P.G., Jayapalan C.S., Hafiz K.A. (2014). Microleakage in endodontics. J. Int. Oral Health.

[4] Wu M., Fan B., Wesselink P. (2000). Diminished leakage along root canals filled with gutta-percha without sealer over time: A laboratory study. Int. Endod. J..

[5] Johnson M.E., Stewart G.P., Nielsen C.J., Hatton J.F. (2000). Evaluation of root reinforcement of endodontically treated teeth. Oral Surg. Oral Med. Oral Pathol. Oral Radiol. Endod..

[6] Cohen S., Berman L.H., Blanco L., Bakland L., Kim J.S. (2006). A Demographic Analysis of Vertical Root Fractures. J. Endod..

[7] Patel S., Bhuva B., Bose R. (2022). Present status and future directions: Vertical root fractures in root filled teeth. Int. Endod. J..

[8] Yoshino K., Ito K., Kuroda M., Sugihara N. (2015). Prevalence of vertical root fracture as the reason for tooth extraction in dental clinics. Clin. Oral Investig..

[9] Ferrari M., Vichi A., Fadda G.M., Cagidiaco M.C., Tay F.R., Breschi L., Polimeni A., Goracci C. (2012). A randomized controlled trial of endodontically treated and restored premolars. J. Dent. Res..

[10] Bier C.A., Shemesh H., Tanomaru-Filho M., Wesselink P.R., Wu M.K. (2009). The ability of different nickel-titanium rotary instruments to induce dentinal damage during canal preparation. J. Endod..

[11] Shemesh H., Bier C.A., Wu M.K., Tanomaru-Filho M., Wesselink P.R. (2009). The effects of canal preparation and filling on the incidence of dentinal defects. Int. Endod. J..

[12] Ribeiro F.C., Souza-Gabriel A.E., Marchesan M.A., Alfredo E., Silva-Sousa Y.T., Sousa-Neto M.D. (2008). Influence of different endodontic filling materials on root fracture susceptibility. J. Dent..

[13] Fisher M.A., Berzins D.W., Bahcall J.K. (2007). An in vitro comparison of bond strength of various obturation materials to root canal dentin using a push-out test design. J. Endod..

[14] Phukan A.H., Mathur S., Sandhu M., Sachdev V. (2017). The effect of different root canal sealers on the fracture resistance of endodontically treated teeth-in vitro study. Dent. Res. J..

[15] Donnermeyer D., Urban K., Bürklein S., Schäfer E. (2020). Physico-chemical investigation of endodontic sealers exposed to simulated intracanal heat application: Epoxy resins and zinc oxide-eugenols. Int. Endod. J..

[16] Bodrumlu E. (2008). Biocompatibility of retrograde root filling materials: A review. Aust. Endod. J..

[17] Pelliccioni G.A., Ciapetti G., Cenni E., Granchi D., Nanni M., Pagani S., Giunti A. (2004). Evaluation of osteoblast-like cell response to Proroot MTA (mineral trioxide aggregate) cement. J. Mater. Sci. Mater. Med..

[18] Torabinejad M., Hong C.U., Pitt Ford T.R., Kettering J.D. (1995). Cytotoxicity of four root end filling materials. J. Endod..

[19] Zhu Q., Haglund R., Safavi K.E., Spangberg L.S. (2000). Adhesion of human osteoblasts on root-end filling materials. J. Endod..

[20] An H.J., Yoon H., Jung H.I., Shin D.H., Song M. (2021). Comparison of Obturation Quality after MTA Orthograde Filling with Various Obturation Techniques. J. Clin. Med..

[21] Lim M., Jung C., Shin D.H., Cho Y.B., Song M. (2020). Calcium silicate-based root canal sealers: A literature review. Restor. Dent. Endod..

[22] Gandolfi M.G., Iacono F., Agee K., Siboni F., Tay F., Pashley D.H., Prati C. (2009). Setting time and expansion in different soaking media of experimental accelerated calcium-silicate cements and ProRoot MTA. Oral Surg. Oral Med. Oral Pathol. Oral Radiol. Endod..

[23] Gomes-Filho J.E., Watanabe S., Bernabe P.F., de Moraes Costa M.T. (2009). A mineral trioxide aggregate sealer stimulated mineralization. J. Endod..

[24] Marciano M.A., Duarte M.A., Camilleri J. (2016). Calcium silicate-based sealers: Assessment of physicochemical properties, porosity and hydration. Dent. Mater..

[25] Chybowski E.A., Glickman G.N., Patel Y., Fleury A., Solomon E., He J. (2018). Clinical Outcome of Non-Surgical Root Canal Treatment Using a Single-cone Technique with Endosequence Bioceramic Sealer: A Retrospective Analysis. J. Endod..

[26] Camilleri J., Grech L., Galea K., Keir D., Fenech M., Formosa L.M., Damidot D., Mallia B. (2013). Porosity and root dentine to material interface assessment of calcium silicate-based root-end filling materials. Clin. Oral Investig..

[27] Saw L.-H., Messer H.H. (1995). Root strains associated with different obturation techniques. J. Endod..

[28] Girish K., Mandava J., Chandra R.R., Ravikumar K., Anwarullah A., Athaluri M. (2017). Effect of obturating materials on fracture resistance of simulated immature teeth. J. Conserv. Dent..

[29] Cleghorn B.M., Christie W.H., Dong C.C.S. (2007). The Root and Root Canal Morphology of the Human Mandibular Second Premolar: A Literature Review. J. Endod..

[30] Uzunoglu Ozyurek E., Aktemur Turker S. (2019). Evaluation of fracture resistance of roots-filled with various root canal sealers at different time periods. Eur. Oral Res..

[31] Arola D.D., Gao S., Zhang H., Masri R. (2017). The Tooth: Its Structure and Properties. Dent. Clin. N. Am..

[32] Tavanafar S., Karimpour A., Karimpour H., Mohammed Saleh A., Hamed Saeed M. (2015). Effect of Different Instrumentation Techniques on Vertical Root Fracture Resistance of Endodontically Treated Teeth. J. Dent..

[33] Topcuoglu H.S., Tuncay O., Karatas E., Arslan H., Yeter K. (2013). In vitro fracture resistance of roots obturated with epoxy resin-based, mineral trioxide aggregate-based, and bioceramic root canal sealers. J. Endod..

[34] Al-Hiyasat A.S., Sawallha A.M., Taha N.A. (2023). The effect of sealer type and obturation technique on the fracture resistance of endodontically treated roots. Clin. Oral Investig..

[35] Uzunoglu E., Aktemur S., Uyanik M.O., Durmaz V., Nagas E. (2012). Effect of ethylenediaminetetraacetic acid on root fracture with respect to concentration at different time exposures. J. Endod..

[36] Aslantas E.E., Buzoglu H.D., Altundasar E., Serper A. (2014). Effect of EDTA, sodium hypochlorite, and chlorhexidine gluconate with or without surface modifiers on dentin microhardness. J. Endod..

[37] Prado M., De Lima N.R.B., De Lima C.O., Gusman H., Simão R.A. (2016). Resistance to vertical root fracture of root filled teeth using different conceptual approaches to canal preparation. Int. Endod. J..

[38] Telli C., Gülkan P., Raab W. (1999). Additional studies on the distribution of stresses during vertical compaction of gutta-percha in the root canal. Br. Dent. J..

[39] Ruiz-Linares M., Solana C., Baca P., Arias-Moliz M.T., Ferrer-Luque C.M. (2022). Antibiofilm potential over time of a tricalcium silicate material and its association with sodium diclofenac. Clin. Oral. Investig..

[40] Reyes-Carmona J.F., Felippe M.S., Felippe W.T. (2010). The biomineralization ability of mineral trioxide aggregate and Portland cement on dentin enhances the push-out strength. J. Endod..

[41] Ferrer-Luque C.M., Baca P., Solana C., Rodriguez-Archilla A., Arias-Moliz M.T., Ruiz-Linares M. (2021). Antibiofilm Activity of Diclofenac and Antibiotic Solutions in Endodontic Therapy. J. Endod..

[42] Ruiz-Linares M., Baca P., Arias-Moliz M.T., Ternero F.J., Rodriguez J., Ferrer-Luque C.M. (2019). Antibacterial and antibiofilm activity over time of GuttaFlow Bioseal and AH Plus. Dent. Mater. J..

[43] Osiri S., Banomyong D., Sattabanasuk V., Yanpiset K. (2018). Root Reinforcement after Obturation with Calcium Silicate-based Sealer and Modified Gutta-percha Cone. J. Endod..

[44] Asawaworarit W., Yachor P., Kijsamanmith K., Vongsavan N. (2016). Comparison of the Apical Sealing Ability of Calcium Silicate-Based Sealer and Resin-Based Sealer Using the Fluid-Filtration Technique. Med. Princ. Pract..

[45] Cimpean S.I., Burtea A.L.C., Chiorean R.S., Dudescu M.C., Antoniac A., Robu A., Campian R.S., Timis L.I. (2022). Evaluation of Bond Strength of Four Different Root Canal Sealers. Materials.

[46] Desai S., Chandler N. (2009). The restoration of permanent immature anterior teeth, root filled using MTA: A review. J. Dent..

[47] Harinkhere C., Patni P.M., Jain P., Raghuwanshi S., Pandey P.M., Bilaiya S. (2024). Comparison of the sealing ability amongst orthograde apical plugs of mineral trioxide aggregate plus, mineral trioxide aggregate repair HP, and Biodentine after root resection: A bacterial leakage study. Odontology.

[48] Alsulaimani R.S. (2016). Single-visit endodontic treatment of mature teeth with chronic apical abscesses using mineral trioxide aggregate cement: A randomized clinical trial. BMC Oral Health.

[49] Yoo Y.J., Baek S.H., Kum K.Y., Shon W.J., Woo K.M., Lee W. (2016). Dynamic intratubular biomineralization following root canal obturation with pozzolan-based mineral trioxide aggregate sealer cement. Scanning.

